# Multimorbidity Patterns in a National HIV Survey of South African Youth and Adults

**DOI:** 10.3389/fpubh.2022.862993

**Published:** 2022-04-04

**Authors:** Rifqah Abeeda Roomaney, Brian van Wyk, Annibale Cois, Victoria Pillay-van Wyk

**Affiliations:** ^1^Burden of Disease Research Unit, South African Medical Research Council, Cape Town, South Africa; ^2^School of Public Health, University of the Western Cape, Cape Town, South Africa; ^3^Division of Health Systems and Public Health, Department of Global Health, University of Stellenbosch, Stellenbosch, South Africa

**Keywords:** multimorbidity, disease pattern, latent class analysis (LCAs), prevalence, South Africa, HIV, low and middle income country

## Abstract

**Introduction:**

Information pertaining to multimorbidity is frequently informed by studies from high income countries and it is unclear how these findings relate to low and middle income countries, where the burden of infectious disease is high. South Africa has a quadruple burden of disease which includes a high HIV prevalence and a growing burden of non-communicable diseases. This study aimed to analyse the prevalence and patterns (disease classes or clusters) of multimorbidity in South Africa.

**Methods:**

A secondary analysis of individuals over the age of 15 years who participated in the Fifth South African National HIV Prevalence, Incidence, Behavior and Communication Survey, 2017 (SABSSM 2017) was done. Six disease conditions were identified in the analysis (cancer, diabetes, heart disease, hypertension/high blood pressure, tuberculosis, and HIV). Chi-square tests were used to test for the differences in disease prevalence by sex. Common disease patterns were identified using a latent class analysis.

**Results:**

The sample included 27,896 participants, of which 1,837 had comorbidity or multimorbidity. When taking population-weighting into account, multimorbidity was present in 5.9% (95% CI: 5.4–6.4) of the population The prevalence of multimorbidity tended to be higher among females and increased with age, reaching 21.9% in the oldest age group (70+). The analyses identified seven distinct disease classes in the population. The largest class was “Diabetes and Hypertension” (36.3%), followed by “HIV and Hypertension” (31.0%), and “Heart disease and Hypertension” (14.5%). The four smaller classes were: “HIV, Diabetes, and Heart disease” (6.9%), “TB and HIV” (6.3%), “Hypertension, TB, and Cancer” (2.8%), and “All diseases except HIV” (2.2%).

**Conclusion:**

As the South African population continues to age, the prevalence of multimorbidity is likely to increase which will further impact the health care system. The prevalence of multimorbidity in the population was relatively low but reached up to 20% in the oldest age groups. The largest disease cluster was the combination of diabetes and hypertension; followed by HIV and hypertension. The gains in improving adherence to antiretrovirals amongst treatment-experienced people living with HIV, should be expanded to include compliance with lifestyle/behavioral modifications to blood pressure and glucose control, as well as adherence to anti-hypertension and anti-diabetic medication. There is an urgent need to improve the early diagnosis and treatment of disease in the South African population.

## Introduction

Multimorbidity is defined as the co-existence of two or more chronic diseases in one individual (1). While it is not easy to determine the number of people living with multimorbidity, a large proportion of the global population are believed to be affected by multimorbidity (2). Multimorbidity negatively impacts individuals, health workers, and the health system as a whole (3). At the individual level, people with multimorbidity have reduced chances of survival (4, 5), lower quality of life (6–8), increased healthcare utilization (9, 10) and tend to experience polypharmacy (11). In 2018, the Academy of Medical Sciences (12) acknowledged that multimorbidity was a global research priority as populations are experiencing multimorbidity on a large scale and predictions that the prevalence of multimorbidity is rising. They also highlighted the inadequate nature of research into multimorbidity, especially in low and middle income countries (LMICs).

LMICs are underrepresented in studies of multimorbidity (13, 14), even though the prevalence is estimated to be as high as 30% (2). Countries such as South Africa have a complex disease burden consisting of both communicable and non-communicable diseases (NCDs) (15). South Africa has a high HIV prevalence with an estimated 7.7 million people living with HIV in 2019 (16). In addition, due to the availability of antiretroviral medication, people living with HIV (PLWH) are living to older ages and developing chronic diseases usually seen among older people (17). The burden of NCDs is also increasing, partly attributed to a transition away from traditional food and toward a “western diet” (i.e., more energy-dense, processed foods, more added sugar, fat, and salt) (18), reductions in physical activity (19), and general aging of the population (20). These risk factors left unchecked, together with a challenging socioeconomic environment characterized by poverty, high unemployment and alcohol and substance abuse, could lead to devastating increases in the incidence of NCDs (21). As the epidemics of NCDs and HIV continue to collide (22), it is important to gain insight into the extent of multimorbidity and the types of multimorbidity present.

Due to the high HIV prevalence in South Africa, a national HIV survey is undertaken approximately every 5 years. This survey aims to describe trends in prevalence and incidence of HIV, as well as to monitor other important self-reported health and behavioral indicators. In this paper, we report on the prevalence of multimorbidity and common disease patterns in the adult South African population from a secondary analysis of the Fifth South African National HIV Prevalence, Incidence, Behavior and Communication Survey (SABSSM 2017).

## Materials and Methods

### Sample and Data Collection

The nationally representative survey aimed to provide surveillance information on HIV infection and behavior, to evaluate progress toward the South African National HIV, AIDS and STI Strategic Plan for 2012–2016, and to provide data used for HIV indicators for national and international bodies (23). The 2017 SABSSM survey was a cross-sectional, population-based household survey that used multi-stage stratified random cluster sampling (23). The survey used the 2015 national sampling frame developed by Statistics South Africa and drew 1,000 small area layers, disproportionally allocated across province (23). Within the small areas, 15 visiting points were randomly selected and all individuals in the household were invited to participate (23). Sampling and design details have been described elsewhere (23).

Fieldworkers collected data from participants. For participants aged 15 years and older, a questionnaire was used that focused mostly on sexual health and behaviors. This questionnaire also contained self-report questions about the presence of several diseases. For these self-reported disease conditions, participants were asked “*Do you currently have any of the following illnesses?*” (Supplementary Table 1). In addition, participants were asked to provide a blood sample to test for HIV status. The blood sample was taken using a finger-prick. These samples were then tested for the presence of the virus at accredited laboratories (23). An algorithm was used with three different enzyme immunoassays (EIAs) whereby if samples tested positive for the HIV during the first two enzyme immunoassays (Roche Elecys HIV Ag/Ab assay, Roche Diagnostics, Mannheim, Germany; and Genescreen Ultra HIV Ag/Ab assay, Bio-Rad Laboratories, California, USA), a third nucleic acid amplification test was done (COBAS AmpliPrep/Cobas Taqman HIV-1 Qualitative Test, v2.0, Roche Molecular Systems, New Jersey, USA) (23). The results of these tests were then interpreted (23).

### Variables of Interest

We considered participants to have multimorbidity if they reported more than one disease condition. We included six disease conditions in our assessment: five were self-report diseases (e.g., cancer, diabetes, heart disease, hypertension/high blood pressure, and TB) and one was the result of a biomarker (HIV status). Although HIV was included in the list of self-reported diseases, we opted to use the result of the HIV biomarker. Of the 27,896 youth and adults that responded to the questionnaire, only 69.9% (*n* = 19,511) had results for HIV testing. Those that did not provide blood specimens or had invalid HIV tests (*n* = 8,385) were set to missing for the HIV variable.

### Analysis

The statistical analysis was done using STATA 15.0 software (Stata Corporation, College Station, Texas, USA). The STATA survey set (“svy”) suite of commands was used to account for complex survey design and the data were weighted using the HIV specimen survey weights. The latent class analysis was conducted using the LCA Stata Plugin which also accounts for complex survey design (24, 25).

An index variable was created to summarize the number of diseases present in each individual. Unweighted sample data were explored using frequency tables. Bivariate associations were assessed using Pearsons's chi-square tests to test for differences among the sexes. The Wilcoxon Rank Sum Test was used to calculate the median age between the sexes.

A sub-analysis was conducted for only people with multimorbidity, to identify the types of disease clustering present. Disease clustering was assessed with LCA; a statistical procedure used to identify sub-groups or classes within populations (26). The LCA followed a process as described by Weller et al. (26). The six disease conditions were included as indicator variables and each disease condition was coded as a binary variable (i.e., disease present or disease absent). To identify latent classes, we first estimated a one-class model and then added additional classes to compare the relative fit (26). We assessed the relative fit of models by comparing the values of a series of information indices [namely the Bayesian information criterion (BIC) (27), the adjusted BIC (aBIC) (28), and the Akaike Information Criterion (AIC) (29)], with lower values indicating a better fit (30). Once the appropriate model was selected, individuals were assigned to the class with the highest posterior probability.

### Ethical Considerations

This study was a secondary data analysis of an anonymized dataset. The anonymized dataset was obtained from the Data Curation Services at the Human Sciences Research Council with necessary permissions. Ethics clearance to conduct this study was granted by the Biomedical Research Ethics Committee of the University of the Western Cape (BM20/5/8).

## Results

### Sample Description (Unweighted Data)

The sample consisted of 27,896 participants: with more females (58.9%) than males (Table 1). The median age of participants was 33 years (IQR 22–50 years) with females tending to be slightly older than males (34 vs. 31 years of median age, *p* < 0.001). A slightly larger proportion of participants lived in urban areas (54.5%) compared to rural areas. The sample was mostly from KwaZulu-Natal (33.9%), Gauteng (15.6%), and Mpumalanga (12.9%) provinces.

**Table 1:** Description of sample by sex (unweighted).

**Variable**	**% (*n*)**			*P*-value^*^
	**Total** **(*N* = 27,896)**	**Male** **(*n* = 11,456)**	**Female** **(*n* = 16,422)**	
Age	33 (22-50)	31 (21-48)	34 (23-51)	<0.001
(Median years and IQR)				
Urban location	54.5 (15,203)	55.5 (6,359)	53.8 (8,839)	0.005
**Province**				<0.001
Western Cape	7.6 (2,107)	7.7 (885)	7.4 (1,222)	
Eastern Cape	7.4 (2,074)	7.3 (831)	7.6 (1,243)	
Northern Cape	5.5 (1,522)	5.9 (679)	5.1 (839)	
Free State	4.4 (1,226)	4.6 (527)	4.3 (698)	
KwaZulu-Natal	33.9 (9,469)	31.3 (3,590)	35.8 (5,874)	
North West	6.6 (1,826)	6.8 (779)	6.4 (1,046)	
Gauteng	15.6 (4,339)	16.5 (1,885)	14.9 (2,453)	
Mpumalanga	12.9 (3,598)	13.9 (1,589)	12.2 (2,006)	
Limpopo	6.2 (1,735)	6.0 (691)	6.3 (1,017)	
**Education level**				<0.001
Primary or less	20.6 (3,832)	18.9 (1,433)	21.8 (2,399)	
Secondary complete	66.8 (12,400)	67.3 (5,097)	66.4 (7,303)	
Tertiary	12.6 (2,344)	13.8 (1,047)	11.8 (1,297)	
Employed	29.5 (7,793)	37.6 (4,092)	23.8 (3,701)	<0.001

* *Chi-square tests used, and Wilcoxon rank-sum test used for Age variable. There were 14 observations with missing data for sex*.

Less than a third of participants in the sample were employed (29.5%); with most being either unemployed, unable to work due to a disability or students. Males were significantly more likely to be employed compared to females (*p* < 0.001). Approximately 67% of the sample had completed secondary education. In the sample, other than TB, all disease conditions were more prevalent in females than males (Supplementary Table 2). The prevalence of multimorbidity in the sample was 6.6% (Supplementary Table 3).

### Disease Prevalence in the Population (Weighted Data)

All percentages from this point forward are weighted unless stated otherwise. HIV was estimated to be the most prevalent disease in the population (19.1%, 95% CI: 17.9–20.4) (Table 2). Hypertension was estimated to be the most second most common disease (14.3%, 95% CI: 13.5–15.2), followed by diabetes (4.4%, 95% CI: 4.0–4.9). Most of the diseases were more prevalent in females compared to males; the only exception was TB which was more common in males (1.3%, 95% CI: 1.0–1.8) than females (1.0%, 95% CI: 0.8–1.4). The prevalence of self-reported disease conditions may have been under-estimated due to participants not knowing whether they had a disease or not wanting to disclose their disease status. For example, among individuals with non-missing HIV test, the estimated prevalence of biomarker HIV (19.1%) was more than double the prevalence of self-reported HIV (6.6%).

**Table 2:** Estimated prevalence of single disease conditions (weighted).

**Disease condition**	**Weighted % (95% CI)**		
	**Total**	**Male**	**Female**
Cancer	0.6 (0.5–0.8)	0.4 (0.3–0.7)	0.8 (0.6–1.1)
Diabetes	4.4 (4.0–4.9)	3.7 (3.2–4.3)	5.0 (4.5–5.6)
Heart disease	2.2 (1.8–2.5)	1.6 (1.3–2.1)	2.6 (2.1–3.2)
HIV	19.1 (17.9–20.4)	14.3 (12.9–15.9)	23.1 (21.7–24.7)
Hypertension	14.3 (13.5–15.2)	10.4 (9.4–11.5)	17.8 (16.7–19.0)
TB	1.2 (1.0–1.4)	1.3 (1.0–1.8)	1.0 (0.8–1.4)

**Figure 1:**
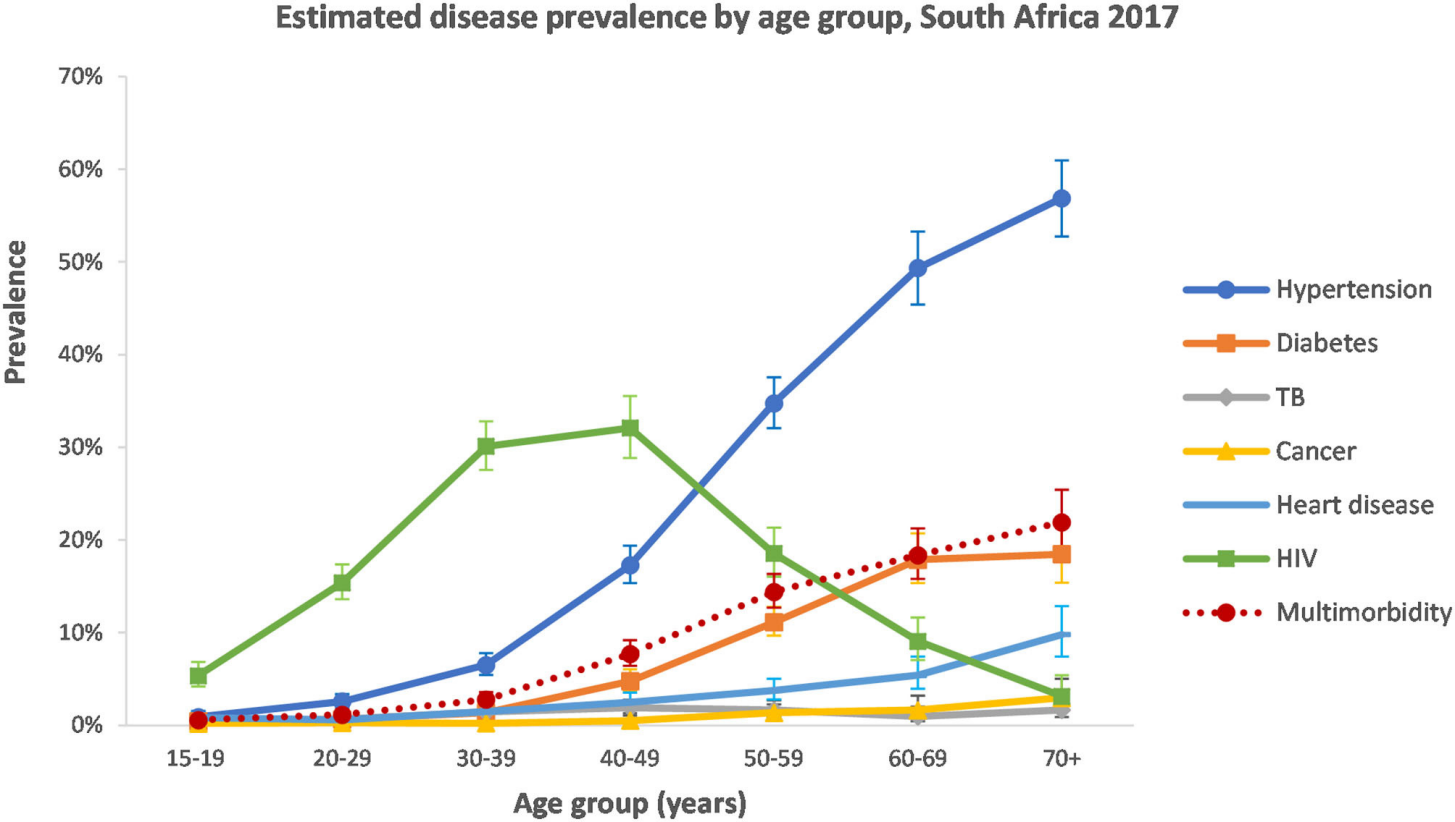
Estimated disease prevalence by age group in South Africa, 2017 (weighted).

With the exception of TB and cancer, the estimated prevalence of disease conditions varied largely by age group (Figure 1). Hypertension, diabetes, and heart disease all increased in the older age groups. HIV followed a different pattern and peaked in the 30–49-year age group.

The number of diseases present in an individual ranged from zero to six (Table 3). Of the diseases assessed in this study, the majority of population had no diseases (71.9%, 95% CI: 70.8–73.1). There were fewer females with no diseases (65.9%, 95% CI: 64.3–67.4) compared to males with no diseases (78.6, 95% CI: 77.3–67.4). A further 22.8% (95% CI: 21.8–23.7) of the population had one disease. Multimorbidity was present in 5.9% (95% CI: 5.4–6.4) of the population. It was more prevalent in females (7.5%, 95% CI: 6.8–8.2) than males (4.1%, 95% CI: 3.6–4.7).

**Table 3:** Estimated number of diseases in individuals by sex (weighted).

**Number of diseases**	**Weighted % (95% CI)**		
	**Total**	**Male**	**Female**
No diseases	71.9 (70.8–73.1)	78.6 (77.3–79.9)	65.9 (64.3–67.4)
1 disease	22.2 (21.2–23.2)	17.3 (16.1–18.5)	26.7 (25.4–28.0)
2 diseases	4.9 (4.5–5.4)	3.5 (3.0–4.0)	6.3 (5.7–6.9)
3 diseases	0.7 (0.6–0.9)	0.4 (0.3–0.6)	1.0 (0.8–1.3)
4 diseases	0.1 (0.0–0.2)	0.0 (0.0–0.1)	0.1 (0.1–0.3)
5+ diseases	0.1 (0.1–0.2)	0.2 (0.1–0.4)	0.1 (0.0–0.2)
**Multimorbidity (≥2 diseases)**	5.9 (5.4–6.4)	4.1 (3.6–4.7)	7.5 (6.8–8.2)

The prevalence of multimorbidity was low in younger age groups for both males and females (Supplementary Figure 1). It increased in the older age groups, reaching 17.0% (95% CI: 15.6–18.5) in people over 50 years. It peaked in the 70+ years age group at 21.9% (95% CI: 18.8–25.4).

### Disease Patterns in the Multimorbid Population

For the subsequent analysis, only people with multimorbidity were included (*n* = 1,837). The mean age of the population with multimorbidity was 54 years. Most of the population with multimorbidity were female (66.7%, 95% CI: 63.1–70.1).

**Figure 2:**
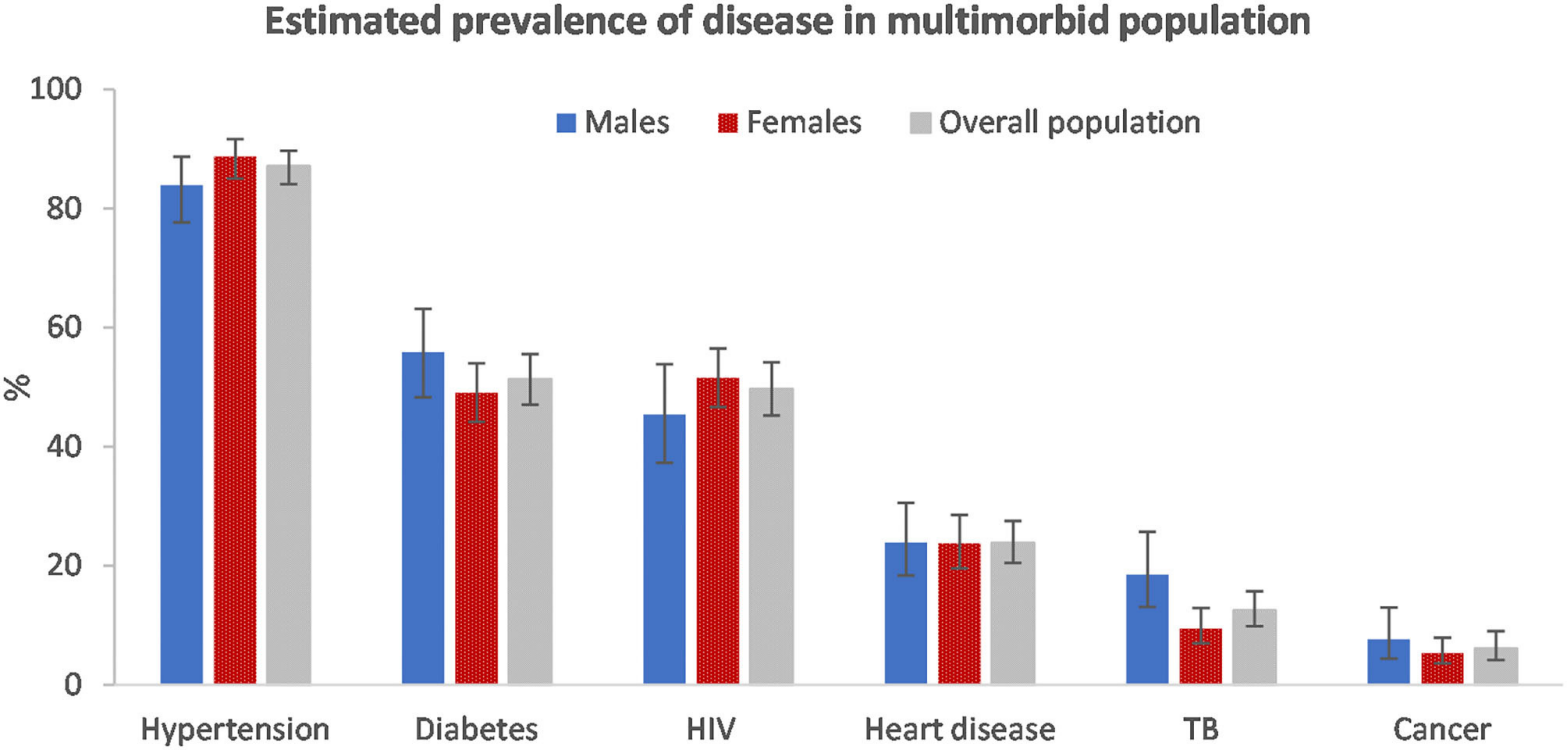
Estimated disease prevalence in the multimorbid population by sex, South Africa 2017.

Among those with multimorbidity, hypertension (87.1%, 95% CI: 84.1–89.7) was the most common disease condition (Figure 2). Just more than half had diabetes (51.3%, 95% CI: 47.0–55.5) and just less than half (49.7%, 95% CI: 45.2–54.1) had HIV. The disease prevalence was similar among males and females with multimorbidity. However, hypertension and HIV were marginally more prevalent among females and, diabetes, TB and cancer were more prevalent among males.

Table 4 shows a comparison of fit statistics for models with different numbers of classes, ranging from two to eight. The BIC, adjusted-BIC and AIC were minimal for a seven class model and hence a seven class model was chosen.

**Table 4:** Fit statistics for LCA models with different numbers of classes.

	**Latent class analysis models**						
	**2 class model**	**3 class model**	**4 class model**	**5 class model**	**6 class model**	**7 class model**	**8 class model**
Design effect	1.9	1.5	1.4	1.3	1.3	1.0	0.9
DF	50.0	43.0	36.0	29.0	22.0	15.0	8.0
Entropy R-squared	0.8	0.8	0.9	0.9	0.9	0.9	0.9
Entropy raw	128.3	220.7	111.2	196.7	300.0	155.5	188.5
Adjusted BIC	1,037.7	736.3	753.7	479.8	464.4	368.0	386.7
BIC	1,079.0	799.8	839.5	587.8	594.7	520.5	561.4
AIC	1,012.6	697.8	701.6	414.3	385.4	275.5	280.6
G-squared	986.6	657.8	647.6	346.3	303.4	179.5	170.6
Log likelihood	−3,084.2	−2,919.8	−2,914.7	−2,764.0	−2,742.6	−2,680.6	−2,676.2

**Figure 3:**
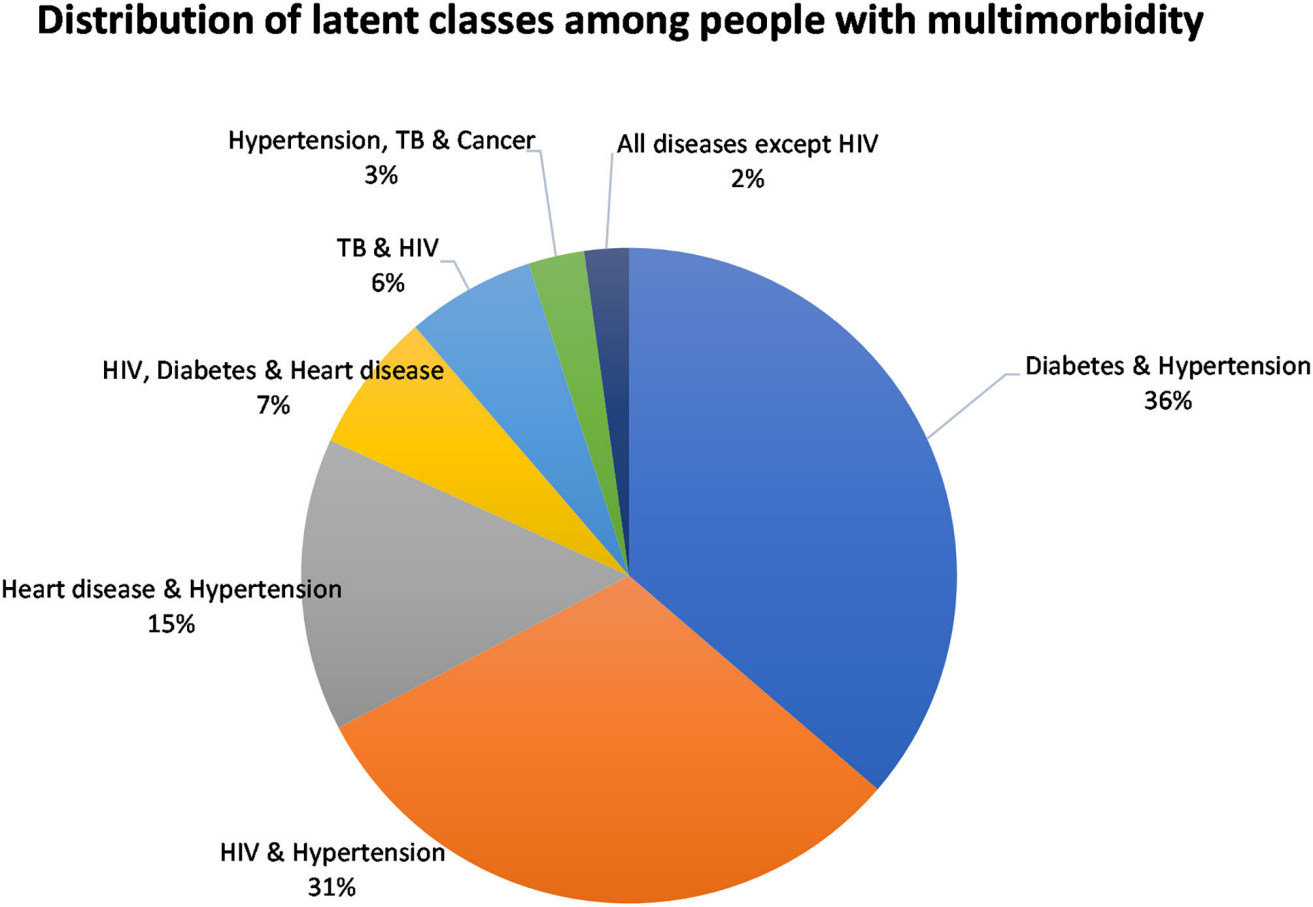
Distribution of latent class membership for people with multimorbidity.

The classes were named based on the diseases with the highest prevalence in that class. The model identified the following membership, latent classes, from largest to smallest: “Diabetes and Hypertension” (36.3%), “HIV and Hypertension” (31.0%), “Heart disease and Hypertension” (14.5%), “HIV, Diabetes and Heart disease” (6.9%), “TB and HIV” (6.3%), “Hypertension, TB, and Cancer,” and “All diseases except HIV” (2.2%) (Figure 3). Hypertension was common among most of the disease classes.

Table 5 displays the item response probabilities for each disease by latent class. The largest class among multimorbid people, “Diabetes and Hypertension,” was characterized by high probabilities of having both diabetes and hypertension (100%). Similarly, the “HIV and Hypertension” class was also a large class among people with multimorbidity and was characterized by a high probability of both HIV and hypertension (100%). The third largest class, “Heart disease and Hypertension” had high probabilities of heart disease and hypertension (100 and 97%, respectively).

**Table 5:** Item response probabilities by disease for each latent class.

**Latent class name**	**Probability of disease condition by class**					
	**Hypertension**	**Diabetes**	**HIV**	**Heart disease**	**TB**	**Cancer**
1. Diabetes and Hypertension	1.00	1.00	0.00	0.02	0.01	0.02
2. HIV and Hypertension	1.00	0.15	1.00	0.07	0.06	0.03
3. Heart disease and Hypertension	1.00	0.30	0.01	1.00	0.00	0.01
4. HIV, Diabetes and Heart disease	0.04	0.54	0.90	0.51	0.00	0.07
5. TB and HIV	0.01	0.01	0.99	0.11	1.00	0.00
6. Hypertension, TB, and Cancer	1.00	0.01	0.01	0.00	0.63	0.49
7. All diseases except HIV	1.00	1.00	0.03	1.00	0.98	0.96

The remaining four classes were small and, together, only accounted for 18.2% of the multimorbid population. The “HIV, Diabetes, and Heart disease” class had a high probability of having HIV (90%) and smaller probabilities of diabetes and heart disease (54 and 51%, respectively). The “TB and HIV” class had high probabilities of TB and HIV (100 and 99%, respectively). The “Hypertension, TB, and Cancer” class was characterized by a 100% probability of hypertension and smaller probabilities of TB and cancer (63.0 and 49%, respectively). The smallest latent class had high probabilities of all diseases except for HIV (i.e., high probabilities for hypertension, diabetes, heart disease, TB, and cancer). The standard errors for the membership probabilities and disease item response probabilities can be found in Supplementary Tables 4, 5.

## Discussion

This study indicated that while there was a fairly low prevalence of multimorbidity among South African youth and adults, the prevalence of multimorbidity increased with age, as is consistent with other studies (2, 31). The prevalence of multimorbidity among older individuals is concerning, especially given that South Africa's population of over-60s is expected to double between 2012 and 2050 (32). This highlights the urgent need for integrated care in the country. Integrated care is considered an essential component in the United Nations Decade of Healthy Aging (2021–2030).

Women had a higher prevalence of multimorbidity compared to men, and this was true across age groups. This gender differential has been found in other local (31) and international studies (2, 33, 34). The differential could be attributed to women being more likely to share their conditions in self-reports; which could be attributed to a mix of biological factors and societal gender inequalities which contribute to the size of the gender gap in self-reported health (35). Women may also be more likely to know their health status as they tend to have higher healthcare utilization compared to men (36, 37). These gender differentials need to be further unpacked to provide insight on how to make health systems more responsive to the needs of men and women with multimorbidity.

The prevalence of multimorbidity estimated in this study is lower than what was estimated by a meta-analysis of multimorbidity prevalence for LMICs (30%) (2). It was also lower when compared to another South African national survey. In the 2007, WHO Study on global AGEing and adult health survey (38) the multimorbidity prevalence was estimated to be 63.4% in people over 50 years of age whereas in our study the prevalence was far lower, around 17% in people over 50 years of age. Compared to the 2003 World Health survey (39), we estimated a similar prevalence in 18–49-year olds (3.0 vs. 5.0%), but a lower prevalence in older ages (50–64 year olds: 14.5 vs. 21.6%, 65+ year age groups: 19.8 vs. 30.1%). However, the estimates of a low multimorbidity prevalence are similar to findings from another national panel survey, the 2008 and 2012 National Income Dynamic Survey (40, 41). This highlights the complexity in trying to compare multimorbidity prevalence estimates across studies. These studies vary in the number of diseases evaluated, the different disease conditions included and the method of data collection used.

The lower prevalence of multimorbidity observed in this survey could be due to various factors. Firstly, the reported prevalence from self-reported disease conditions was low. This is especially true for hypertension, which is reported to have a higher prevalence in the country (46% women and 44% men) vs. 14% in this survey (42). Similarly, our estimate for diabetes was 4%, while a recent systematic review reported the prevalence of diabetes in South Africa to be close to 15% for people aged 25 years and older (43). The lower multimorbidity prevalence may be linked to how questions about self-reported disease conditions were asked. In this survey, participants were asked if they “currently” had the disease in question. In many other surveys, participants are asked if they have “ever” been diagnosed with a set of diseases. The incorporation of “current” disease is likely to be more accurate compared to those who have “ever” been diagnosed. For example, people that have been diagnosed with cancer during their lifetime may have recovered and may be considered disease-free. For future surveys that are conducted, we recommend that participants are asked if they have ever had a disease and whether they currently have the disease. This should lead to more accurate estimations of multimorbidity and allow for data to be more easily compared between surveys, however, problems with underreporting and under-diagnosis will most likely continue to persist in self-report data.

We had a mix of concordant and discordant multimorbidity disease latent classes in our study. Concordant multimorbidity tends to be similar in its origin or etiology, whereas discordant multimorbidity is when the co-existing disease conditions tend to be unrelated (12). A local study defined concordant multimorbidity as hypertension or diabetes with other cardiometabolic conditions (dyslipidemia, angina) (44). They determined discordant conditions to be HIV with any of the following: hypertension/diabetes/angina/dyslipidemia. Using their conceptualization, we would consider the following classes in our study to be concordant: “Diabetes and Hypertension” and “Heart disease and Hypertension.” The largest disease class in this study was the combination of hypertension and diabetes. The clustering of diabetes and hypertension is well-known and has been termed the “bad companions” (45). Their pathophysiological trajectories are interlinked (45). The “HIV and Hypertension” class is an example of discordant multimorbidity and was the second largest class in our analysis. These diseases frequently co-occur, especially in ART experienced individuals (46). Also, this disease combination is predicted to increase as more people access ART and initiate earlier; thereby improving survival rates (46). The effect of discordant vs. concordant multimorbidity on treatment outcomes is unclear.

To reduce, detect and manage multimorbidity a multifactorial approach is required. The South African government has made some progress regarding the management of multimorbidity, as exemplified in the release of the South African National Department of Health Adherence Guidelines for HIV, TB and NCDs in 2016 (47). This policy and service delivery guidelines seek to address issues in non-adherence to long-term therapies amidst the expansion of ART programmes and the rising burden of NCDs (47). Certain aspects of the programme implementation related to this policy have been positively evaluated (48, 49). Another part of their strategy was to focus on linkage to care and to implement screening activities to identify diseases early for intervention. These guidelines complement other South African guidelines and strategies that have been put in place to reduce disease burdens. These include the Strategic Plan for the Prevention and Control of Non-Communicable Diseases 2013–2017 (50), the Strategy for the Prevention and Control of Obesity in South Africa 2015–2020 (51) and, legislation to decrease sodium levels in the food industry (52).

### Limitations

This study entailed a secondary analysis of survey data and was thus limited to the data reported in the survey. We used a combination of biomarker and self-report data which most likely led to a downward bias in our estimate of multimorbidity prevalence. The prevalence of the self-reported disease conditions was lower than expected compared to other local studies that have used biomarker data. This indicates that participants may have been unaware that they have the disease or may not have disclosed that they have the disease for some other reason. This underestimation of self-reported disease has resulted in a reduced estimate of multimorbidity prevalence in the population. In addition, the number of people that provided blood specimens for HIV testing was lower than the number of people that completed the questionnaire and answered questions about self-reported disease conditions. We also included only a few disease conditions which may have also led to an underestimation in the prevalence of multimorbidity.

## Conclusion

This is the first analysis of multimorbidity and disease patterns from a nationally representative HIV survey of South African youth and adults. This study found a relatively low level of multimorbidity prevalence amongst youth and adults in South Africa. The prevalence of multimorbidity increased with age and was also higher in females compared to males. We identified seven classes (sub-groups) for those with multimorbidity, and there was a mix between concordant and discordant disease conditions. The “Diabetes and Hypertension-related” class was the largest, indicating that far more needs to be done to reduce the common causal factors for these diseases. This is an example of concordant multimorbidity; which indicates that they share common risk factors which can be targeted for intervention. The second-largest class was an example of discordant multimorbidity, the combination of HIV and hypertension. Large-scale implementation of the Adherence Guidelines for HIV, TB and NCDs would help to reduce the impact of multimorbidity.

## Data Availability Statement

The original contributions presented in the study are included in the article/Supplementary Material, further inquiries can be directed to the corresponding author.

## Ethics Statement

This study was a secondary data analysis of an anonymized dataset. The anonymized dataset was obtained from the Data Curation Services at the Human Sciences Research Council with necessary permissions. Ethics clearance to conduct this study was granted by the Biomedical Research Ethics Committee of the University of the Western Cape as part of the author's PhD study (BM20/5/8). Written informed consent to participate in this study was provided by the participants' legal guardian/next of kin.

## Author Contributions

RR drafted the first version of the manuscript and conducted data analysis and it was overseen by AC. All authors conceptualized the manuscript, contributed to revising the manuscript, and approved the final version.

## Funding

This work reported herein was made possible through funding by the Burden of Disease Research Unit at the South African Medical Research Council. RR conducted this research under the South African Medical Research Council through its Division of Research Capacity Development under the Internship Scholarship Programme from funding received from the South African National Treasury.

## Author Disclaimer

The content hereof is the sole responsibility of the authors and does not necessarily represent the official views of the South African Medical Research Council or the funders.

## Conflict of Interest

The authors declare that the research was conducted in the absence of any commercial or financial relationships that could be construed as a potential conflict of interest.

## Publisher's Note

All claims expressed in this article are solely those of the authors and do not necessarily represent those of their affiliated organizations, or those of the publisher, the editors and the reviewers. Any product that may be evaluated in this article, or claim that may be made by its manufacturer, is not guaranteed or endorsed by the publisher.
