# Supplementary material for: Multimorbidity Patterns in a National HIV Survey of South African Youth and Adults
Source: Front Public Health. 2022 Apr 4;10:862993. doi: 10.3389/fpubh.2022.862993 (PMC9015099; doi:10.3389/fpubh.2022.862993)
Supplement: Supplementary file 1 [file Data_Sheet_1.docx]

Supplementary Material

**Supplementary Table 1. Questions on self-reported disease conditions from the 2017 SABSSM Adult and Youth Questionnaire (15 years and older).**

| **Disease condition** | **Question** |
| --- | --- |
| Cancer | Do you currently have any of the following illnesses? (Diagnosed with illness) Cancer |
| Diabetes or blood sugar | Do you currently have any of the following illnesses? (Diagnosed with illness) Diabetes |
| Heart disease | Do you currently have any of the following illnesses? (Diagnosed with illness) Heart disease |
| High blood pressure | Do you currently have any of the following illnesses? (Diagnosed with illness) Hypertension / high blood pressure |
| HIV | Do you currently have any of the following illnesses? (Diagnosed with illness) HIV |
| Tuberculosis | Do you currently have any of the following illnesses? (Diagnosed with illness) Tuberculosis/ TB |

**Supplementary Table 2. Prevalence of single disease conditions by sex in the sample (unweighted data).**

| **Disease condition** | **Unweighted % (n/N)*** | | | |
| --- | --- | --- | --- | --- |
|  | **Total** | **Male** | **Female** | **P-value** |
| Cancer | 0.6  (149 / 26 551) | 0.4  (46 / 10 923) | 0.7  (103 / 15 628) | 0.011 |
| Diabetes | 5.5  (1 455 / 26 568) | 4.4  (479 / 10 929) | 6.2  (976 / 15 639) | <0.001 |
| Heart disease | 2.1  (563 / 26 541) | 1.8  (201 / 10 918) | 2.3  (362 / 15 623) | 0.008 |
| Hypertension | 15.9  (4 223 / 26 562) | 11.1  (1 208 / 10 927) | 19.3  (3 015 / 15 635) | <0.001 |
| HIV (Self-report) | 6.4  (1 692 / 26 458) | 3.9  (424 / 10 874) | 8.1  (1 268 / 15 584) | <0.001 |
| HIV (Biomarker) | 19.3  (3 755 / 19 511) | 13.3  (998 /7 517) | 23.0  (2 754 / 11 980) | <0.001 |
| TB | 1.0  (262 / 26 558) | 1.2  (129 / 10 924) | 0.9  (133 / 15 634) | 0.007 |

***n/N = Number of observations/ Total**

**Supplementary Table 3. Number of diseases in individuals by sex in the sample (unweighted data).**

| **Number of diseases** | **Unweighted % (n)** | | | |
| --- | --- | --- | --- | --- |
|  | **Total**  **(N = 27 896)** | **Male**  **(n = 11 456)** | **Female**  **(n = 16 422)** | **P-value** |
| No diseases | 70.7 (19 722) | 79.1 (9 056) | 64.9 (10 651) | <0.001 |
| 1 disease | 22.7 (6 337) | 16.3 (1 867) | 27.2 (4 467) |  |
| 2 diseases | 5.5 (1 544) | 3.9 (444) | 6.7 (1 100) |  |
| 3 diseases | 0.8 (232) | 0.6 (67) | 1.0 (165) |  |
| 4 diseases | 0.08 (22) | 0.1 (6) | 0.1 (16) |  |
| 5+ diseases | 0.14 (39) | 0.2 (16) | 0.1 (23) |  |
| **Multimorbidity**  **(≥ 2 diseases)** | **6.6**  **(1 837 / 27 896)** | **4.7**  **(533 / 11 456)** | **7.4**  **(1 304 / 16 422)** | **<0.001** |

**Supplementary Table 4. Estimated membership probabilities and standard errors for each latent class**

| **Latent class name** | **Membership probability** | **Standard error** |
| --- | --- | --- |
| 1. Diabetes & Hypertension | 0.3630 | 0.022 |
| 1. HIV & Hypertension | 0.3101 | 0.022 |
| 1. Heart disease & Hypertension | 0.1446 | 0.015 |
| 1. HIV, Diabetes & Heart disease | 0.0693 | 0.012 |
| 1. TB & HIV | 0.0634 | 0.011 |
| 1. Hypertension, TB & Cancer | 0.0276 | 0.007 |
| 1. All diseases except HIV | 0.0219 | 0.007 |

**Table 5. Item response probabilities and standard error by disease for each latent class.**

| **Latent class name** | **Item response probability of disease condition by class (Standard error)** | | | | | |
| --- | --- | --- | --- | --- | --- | --- |
|  | **Hypertension** | **Diabetes** | **HIV** | **Heart disease** | **TB** | **Cancer** |
| 1. Diabetes & Hypertension | 1.000  (0.000) | 1.000  (0.000) | 0.003  (0.004) | 0.015  (0.024) | 0.010  (0.005) | 0.025  (0.013) |
| 1. HIV & Hypertension | 1.000  (0.000) | 0.149  (0.030) | 1.000  (0.000) | 0.075  (0.027) | 0.060  (0.019) | 0.033  (0.019) |
| 1. Heart disease & Hypertension | 0.999  (0.002) | 0.300  (0.070) | 0.011  (0.023) | 0.999  (0.000) | 0.000  (0.000) | 0.015  (0.011) |
| 1. HIV, Diabetes & Heart disease | 0.040  (0.059) | 0.543  (0.094) | 0.899  (0.036) | 0.507  (0.092) | 0.001  (0.000) | 0.069  (0.031) |
| 1. TB & HIV | 0.015  (0.015) | 0.006  (0.005) | 0.986  (0.013) | 0.113  (0.058) | 0.998  (0.001) | 0.000  (0.000) |
| 1. Hypertension, TB & Cancer | 0.999  (0.000) | 0.007  (0.002) | 0.006  (0.002) | 0.002  (0.001) | 0.631  (0.142) | 0.485  (0.142) |
| 1. All diseases except HIV | 0.999  (0.000) | 0.997  (0.001) | 0.034  (0.031) | 0.995  (0.002) | 0.985  (0.017) | 0.958  (0.043) |

**Supplementary Figure 1.** **Multimorbidity prevalence by age group and sex (weighted data).**
